# Coagulopathy and Acute Respiratory Distress Syndrome: Dual Complications of E-Cigarette-Associated Lung Injury

**DOI:** 10.7759/cureus.13531

**Published:** 2021-02-24

**Authors:** Atefeh Kalantary, Basel Abdelazeem, Nasheed Shams, Rebecca Pratiti, Ibrahim Al-Sanouri

**Affiliations:** 1 Internal Medicine, McLaren Health Care, Flint/Michigan State University (MSU), Flint, USA; 2 Internal Medicine, McLaren Health Care, Flint, USA; 3 Pulmonary Critical Care, McLaren Flint Hospital/Michigan State University (MSU), Flint, USA

**Keywords:** e-cigarette, vaping, e-cigarette associated lung injury, disseminated intravascular coagulation (dic), acute respiratory distress syndrome (ards)

## Abstract

E-cigarette-associated lung injury (EVALI) is related to the usage of e-cigarettes or a related product (e.g., “vaping” or “dabbing”). It presents mainly with constitutional, respiratory, or gastrointestinal symptoms, and EVALI is currently a diagnosis of exclusion. EVALI patients are more prone to rapid clinical decline requiring close monitoring, with some requiring intensive care unit (ICU) level of care or intubation. Mortality occurs in rare cases.

We are presenting an interesting case of a male in his mid-60s who presented to the emergency department with worsening dyspnea and cough for two weeks, preceded by a one-week history of fever, nausea, and diarrhea. He was diagnosed with bilateral pneumonia based on computed tomography (CT) findings. Subsequent CT of the chest showed worsening bilateral diffuse ground-glass opacities (GGOs) correlating with acute respiratory distress syndrome (ARDS). Laboratory workup showed leukocytosis and lactic acidosis. The rest of the laboratory workup was normal. The patient was intubated due to ARDS, developed multiorgan failure, and passed away subsequently.

## Introduction

Electronic cigarettes (e-cigarettes) aerosolize a variety of products, including glycerol, propylene glycol, nicotine flavoring, or cannabis, which are inhaled by consumers. They are marketed as safer alternatives to tobacco smoking, leading to their increased use and popularity. The potential harm of personalized vaping is being debated in the media and health field, especially given the positive media projection of e-cigarette as having minimal or no health effects, as compared to cigarette smoking. To the best of our knowledge, only a few cases have been reported with coagulopathy and acute respiratory distress syndrome (ARDS) due to e-cigarette-associated lung injury (EVALI). Herein, we present a case and emphasize the importance of taking any history concerning alternate tobacco products, including e-cigarettes or vaping.

## Case presentation

A mid-60s Caucasian male with a past medical history of hypertension, hypercholesterolemia, and hypothyroidism presented to the emergency room (ED) with a one-week history of intermittent fevers associated with mild nausea and diarrhea. He also complained of wheezing and sinus drainage without any cough or shortness of breath.

The social history revealed a pattern of daily marijuana vaping for at least two months prior to presentation. The patient denied any history of cigarette smoking, alcohol, or other drugs. He did not have any history of aspiration, trauma, smoke inhalation, or abnormal occupational exposures that may have caused pneumonitis. There was no history of chemotherapeutic drug exposure or recent surgery.

**Figure 1:**
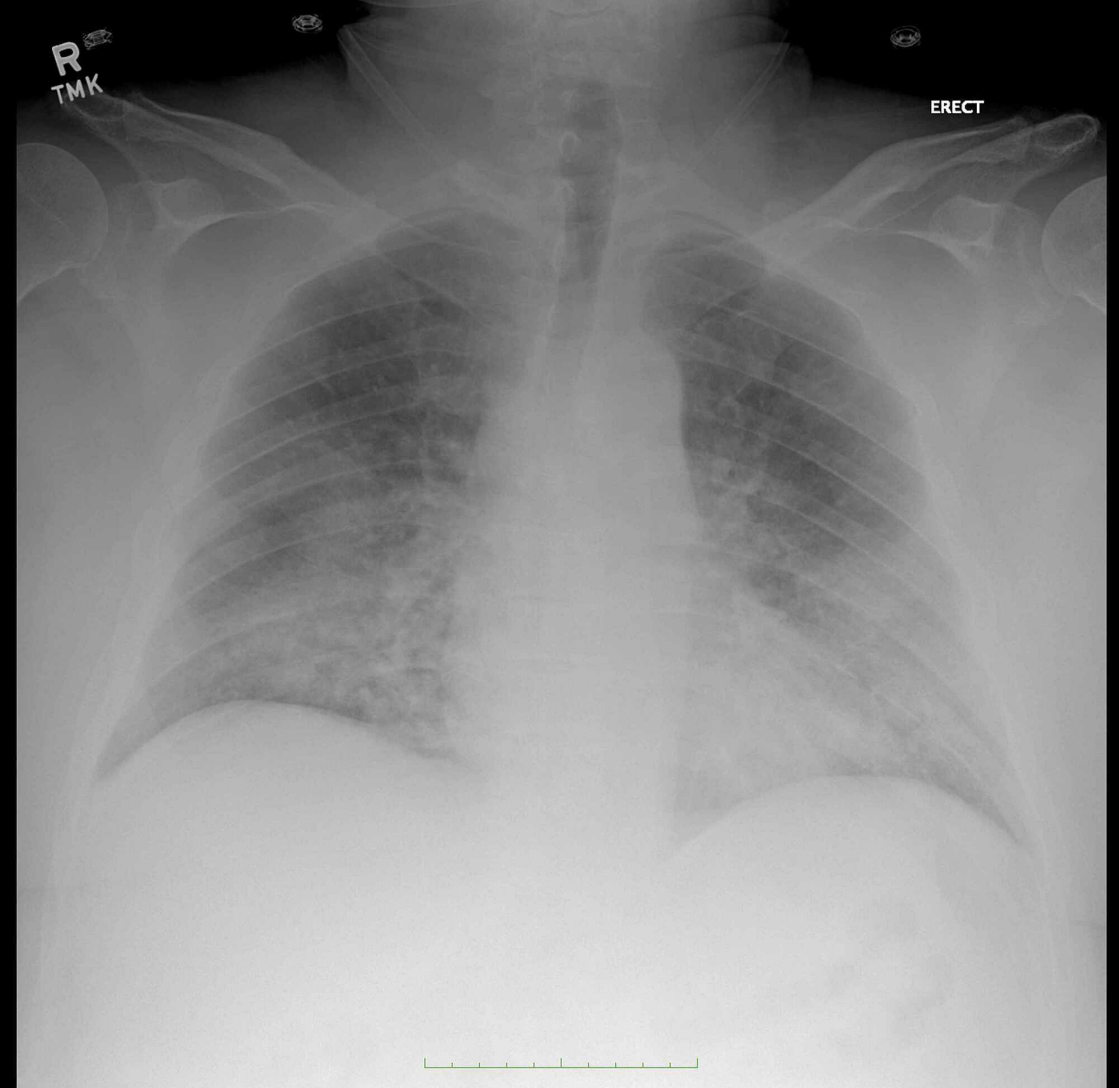
Chest X-ray during the first emergency visit showing bilateral pulmonary infiltrates and bilateral lung airspace disease suggesting edema or pneumonia

**Figure 2:**
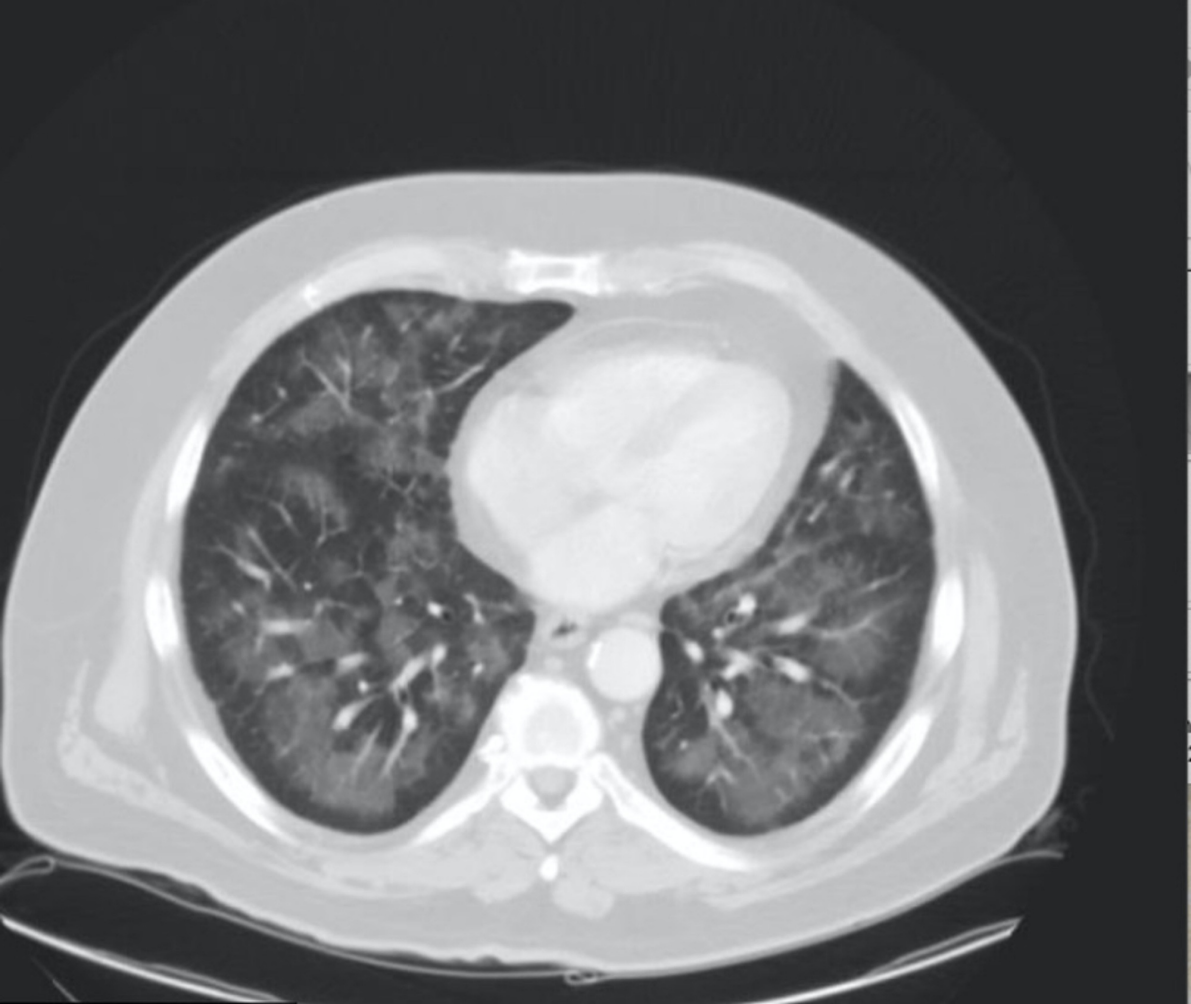
CT chest on the first emergency visit showing bilateral ground-glass opacities, likely pneumonia or edema CT: computed tomography

On examination, his temperature was 101.5 °F, heart rate (HR) 116/minute, and blood pressure (BP) 166/95 mmHg. Bilateral dry crackles were audible on the lung exam. Laboratory results on presentation and during the hospital course are summarized in Table 1. The chest X-ray showed bilateral pulmonary infiltrates (Figure 1). Chest computed tomography (CT) without contrast revealed bilateral ground-glass lung opacities (GGOs) (Figure 2). Bilateral pneumonia was suspected. He declined medical advice to be admitted to the hospital and was discharged on oral antibiotics.

**Table 1:** Laboratory values during the hospitalization ED: emergency department; BUN: blood urea nitrogen; eGFR: estimated glomerular filtration rate; WBC: white blood cell; INR: international normalized ratio

	Reference range	1st ED visit	2nd ED visit - hospital day 1	Hospital day 2	Hospital day 3
BUN (mg/dL)	7-22	11	21	40	62
Creatinine (mg/dL)	0.5-1.5	0.9	1.28	3.01	4.68
eGFR (mL/min)	60-200	NA	58	20	12
Albumin (g/dL)	3.5-5	2.8	2.2	1.4	1.3
Alanine aminotransferase (U/L)	7-36	31	30	361	450
Aspartate aminotransferase (U/L)	8-40	33	47	1769	1442
Alkaline phosphatase (U/L)	39-117		161	129	109
WBC (k/µL)	4.5-11	13.91	21.76	17.88	26.17
Neutrophils (%)		90	90	89	79 + Bands 6%
Lymphocytes (%)		6	6	8	8
Monocytes (%)		2	2	1	1
Eosinophils (%)		1	1	0	1
Basophils (%)		1	1	0	
Myelocyte (%)		0	0	1	1
Metamyelocyte (%)		0	0	1	4
Platelet (X 10*3/uL)	140-440	293	308	44	75
Hemoglobin (g/dL)	13.5-17.7	14.6	15.1	8.9	6.9
INR	0.88-1.11		1.38		
Troponins (ng/mL)	0.00-0.04		0.06	10.73	15.06

**Figure 3:**
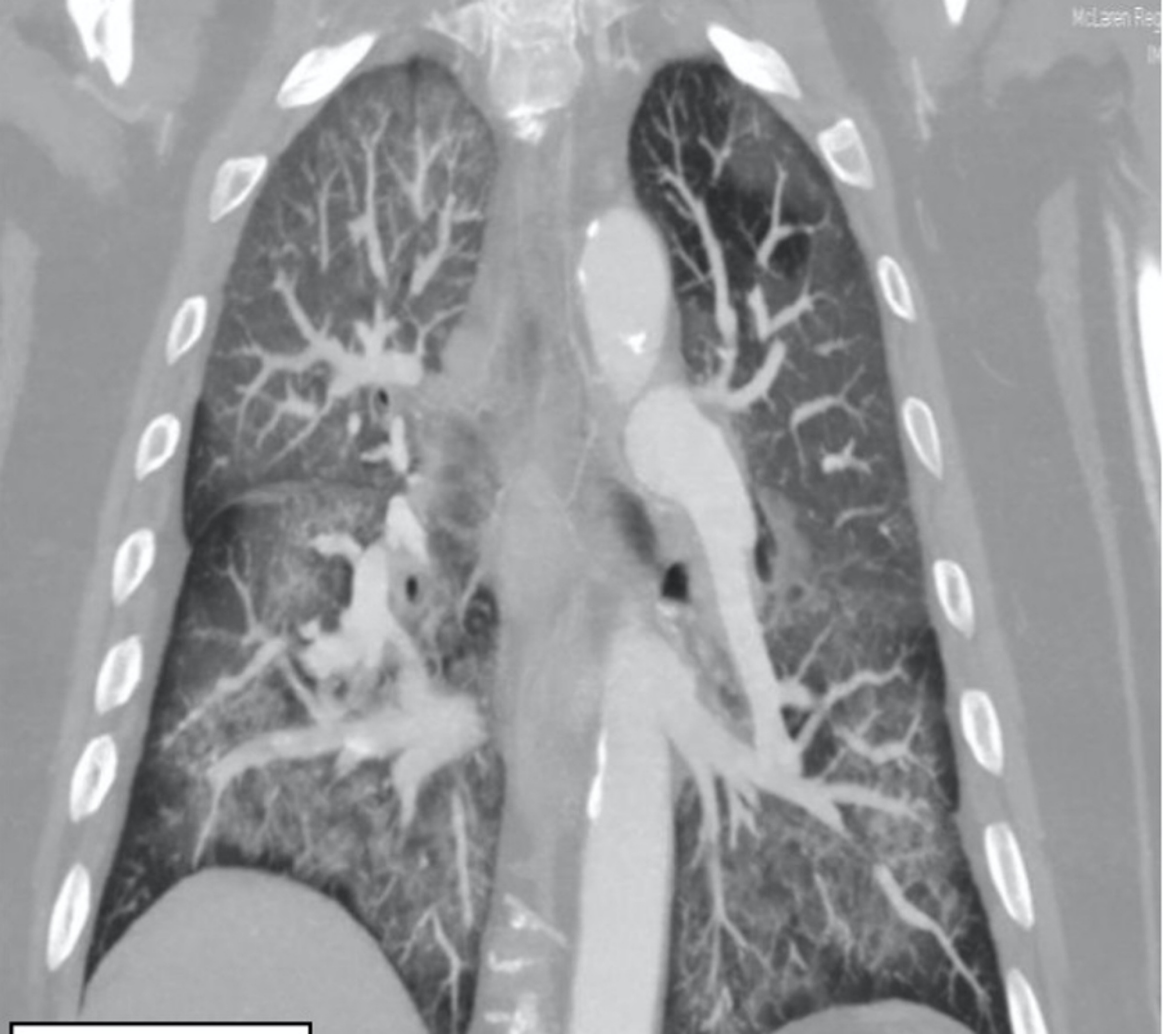
CT chest (coronal view) on second emergency visit showing worsening ground-glass opacities, paratracheal, aorta-pulmonic, and subcarinal lymphadenopathy CT: computed tomography

The patient returned to the emergency department (ED) after five days with severe dyspnea, productive cough, with whitish sputum. His family reported that he continued to have fevers and wheezing at home. The patient saturated 64% on room air, had a respiratory rate of 40 breaths per minute, temperature 98.4 F, HR of 121/min, and BP of 155/65 mmHg. Lung auscultation revealed bilateral expiratory wheezing and dry crackles. Repeat CT of the chest with contrast showed worsening bilateral diffuse ground-glass opacities and subpleural sparing and was negative for pulmonary embolism (Figure 3). Transthoracic echocardiogram revealed an ejection fraction of 45%-50%, with severe right ventricular dilation.

Blood cultures collected before initiating any antibiotics and were negative after five days. Further urinalysis, urine culture, and sputum cultures were negative for infection. A viral respiratory panel, including influenzas A and B polymerase chain reaction (PCR), respiratory syncytial virus (RSV) PCR, adenovirus PCR, rhinovirus PCR, enterovirus, and parainfluenza virus 1, 2, and 3, were negative. Complements C3 and C4 were within a normal range. Antineutrophil cytoplasmic antibodies (ANCA) testing for myeloperoxidase-ANCA and proteinase 3 were negative. Direct and indirect Coombs test was normal. Coronavirus disease 2019 (COVID-19) was not present at that time.

The patient was intubated due to ARDS and started on mechanical ventilatory support and broad-spectrum antibiotics and steroids. Shortly after intubation, the patient developed severe shock (mean arterial pressure of 35 mmHg), and the patient was resuscitated with intravenous fluids and started on pressors.

During his three-day course of hospitalization, his clinical condition deteriorated rapidly. He was noted to have mildly mottled abdominal skin and bilateral cold feet. Distal pedal pulses were non-palpable. Doppler ultrasound, which was performed on admission, showed bilateral severe superficial femoral artery occlusive disease, popliteal, posterior tibial, and dorsalis pedis arteries could not be detected, suggesting occlusion or a consequence of low cardiac output. Vascular surgery was consulted, and the patient was diagnosed with bilateral acute limb ischemia. Surgery was deferred due to the patient’s critical condition, and the patient was started on heparin. Over the next 48 hours, the patient developed ascending mottling of his skin, which progressed to involve his scrotum by the third day. He developed rapidly progressive multiorgan failure, including acute kidney injury, shock liver, thrombocytopenia, and acute right heart failure. The heart failure was attributed to ARDS.

On hospital Day 3, a family meeting was held to discuss the patient’s multiorgan failure and poor prognosis. The family changed the code status to hospice, and the patient expired three hours post-extubation.

## Discussion

People are using e-cigarettes to smoke nicotine, but e-cigarettes can also deliver other substances, such as tetrahydrocannabinol (THC), like our patient [1]. The study by Pacula et al. reported that the median age of EVALI is 24 years, and the median age for the patient who died from EVALI was 54 years [2]. Almost 82% of EVALI patients reported using any TCH-containing e-cigarette or vaping product [1].

Blount et al. reported that 94% of 51 patients exposed to e-cigarettes containing THC had lung injury and a considerable amount of vitamin E acetate in their bronchi alveolar lavage (BAL) fluid [3]. Our patient could not undergo bronchoscopy to confirm the presence of vitamin E acetate in the BAL fluid.

A more recent EVALI case series study done by Ramirez et al. (2020) has suggested inflammatory-coagulation axis derangement in EVALI and increased vitamin E levels antagonizing vitamin K, thereby affecting the clotting cascade [4]. This correlated more with the milder increase in INR seen in our patient. ARDS in itself may cause mild coagulopathy, as well as thrombosis. The etiology for coagulopathy with thrombosis in our patient is uncertain. In our case, disseminated intravascular coagulation (DIC) may have occurred as a complication from ischemic injury to the liver, although the skin findings were immediate and occurred simultaneously or preceded the lab findings of multiorgan failure. However, EVALI-induced coagulopathy and DIC seem likely but further research is needed.

EVALI consists of four patterns of lung disease in CT scans, including acute eosinophilic pneumonia, diffuse alveolar damage, organizing pneumonia, and lipoid pneumonia [5]. In a recent radiologic review of pediatrics patients with EVALI, significant CT chest findings included confluent ground-glass opacities in 100% of patients and subpleural sparing in 75% of patients. Our patient’s CT chest findings showed typical features of EVALI, including confluent ground-glass opacities with frequent subpleural sparing [6].

The patient met EVALI criteria based on 1) use of an e-cigarette or a related product (e.g., “vaping” or “dabbing”) in the previous 90 days; 2) lung opacities on the chest radiograph or CT scan; 3) exclusion of lung infection; 4) absence of a likely alternative diagnosis (e.g., cardiac, neoplastic, rheumatologic) [7-8]. The diagnosis is further supported by his initial presentation of gastrointestinal symptoms preceding the ARDS. EVALI patients are more susceptible to clinical deterioration; 50% of the patients will require admission to the Intensive care unit, 20% will require intubation and mechanical ventilation, and the mortality rate is 2% [9].

## Conclusions

The clinician should be aware of EVALI, which presents dramatically with rapid deterioration in the patient's clinical course. We present this case to highlight the importance of taking any history concerning alternate tobacco products, including e-cigarettes or vaping, given the increased popularity of vaping usage in the adult and elderly population.
